# Educational and emotional health outcomes in adolescence following maltreatment in early childhood: A population-based study of protective factors

**DOI:** 10.1016/j.chiabu.2018.05.008

**Published:** 2018-07

**Authors:** Nisreen Khambati, Liam Mahedy, Jon Heron, Alan Emond

**Affiliations:** Population Health Sciences, Bristol Medical School, University of Bristol, Oakfield House Oakfield Grove, Bristol, BS8 2BN, United Kingdom

**Keywords:** ALSPAC, Avon Longitudinal Study of Parents and Children, WEMWBS, Warwick-Edinburgh Mental Wellbeing Scale, RSE-B, Bachman Self-Esteem Scale, NPD, National Pupil Database, GCSE, General Certificate of Secondary Education, CPR, Child Protection Register, OR, odds ratio, CI, confidence intervals, SEAL, The Social and Emotional Aspects of Learning, ALSPAC, Child maltreatment, Resilience, Protective factors, Education, Wellbeing

## Abstract

Although childhood maltreatment is associated with long-term impairment, some children function well despite this adversity. This study aimed to identify the key protective factors for good educational attainment and positive emotional health in adolescents who experienced maltreatment in early childhood. Data were analyzed from the Avon Longitudinal Study of Parents and Children, a large UK prospective cohort study. The sample was defined by maternally reported exposure to physical or emotional maltreatment by a parent prior to 5 years. 1118 (8.0%) children were emotionally maltreated and 375 (2.7%) were physically maltreated before the age of 5. There were too few cases of sexual abuse to be considered. Positive outcomes were operationalized as achieving 5 or more grade A*-C GCSE exam grades at 16 years and scores above the cohort median on the self-report Warwick-Edinburgh Mental Wellbeing Scale and Bachmann Self-Esteem Scale at 17.5 years. The associations of individual, family and community covariates with successful adaptation to the adversity of maltreatment were investigated using logistic regression.

School related factors, including engagement in extracurricular activities, satisfaction with school and not being bullied were the most important in facilitating resilience in educational attainment, self-esteem and wellbeing. Good communication and social skills was the most protective individual trait. There was insufficient evidence to suggest that family factors were associated with resilience to maltreatment. School-based interventions are recommended to promote positive adaptation following parental maltreatment. Future research should evaluate outcomes across the life-course to understand whether the protective influences of school persist into adulthood.

## 1 Introduction

Child maltreatment – encompassing neglect and the physical, sexual, and emotional abuse of children – is a major public health issue worldwide (Butchart, Putney, Furniss, & Kahane, 2006). In the UK, over 58,000 children were identified as needing protection in 2016 (National Society for the Prevention to Cruelty to Children, 2017), although official figures underestimate the true prevalence. Child maltreatment commonly occurs within the family (Butchart et al., 2006; Longfield, 2015; Radford et al., 2011) and is associated with long-term impairments in multiple domains including physical and mental health problems, poor academic achievement and increased criminal behavior (Gilbert et al., 2009; World Health Organisation, 2016).

Fortunately, many children experiencing maltreatment show evidence of positive adaptation (DuMont, Widom, & Czaja, 2007; Herrenkohl, Tajima, Whitney, & Huang, 2005; Jaffee, Caspi, Moffitt, Polo-Tomás, & Taylor, 2007), with better than expected outcomes suggesting that factors associated with successful adaptation are not simply markers of lower severity of risk. Definitions of resilience vary amongst studies, both in theory and in the operationalization of key constructs and concepts (Afifi & Macmillan, 2011; Walsh, Dawson, & Mattingly, 2010). Most experts consider resilience to be a dynamic and interactive process, encompassing positive adaptation within the context of significant adversity (Luthar, Cicchetti, & Becker, 2000; Rutter, 2012). Studies have documented evidence of uneven adaptation across domains; for example, children exposed to adversity may manifest competence in one area, but experience problems in another (Luthar et al., 2000). Researchers of resilience therefore need to focus on multiple areas of functioning and be specific to which domain is being referred to (Cicchetti & Garmezy, 1993; Walsh et al., 2010).

Mechanisms promoting resilience are context- and culture-dependent, and factors associated with resilience will vary according to the adversity and domain of functioning considered (Luthar et al., 2000; Ungar, 2013b). Two approaches to modelling resilience have been proposed: variable- and person- centered. A variable-centered approach examines statistical associations between measures of adversity, competence and hypothesized protective factors through main effect models and interactive effects; whereas a person-centered approach compares individuals with similar adversity but varying levels of adaptation and identifies differentiating factors associated with individuals meeting predefined criteria for resilience (Masten & Powell, 2003). Whilst the person-centered approach requires larger samples and is less sensitive for identifying explanatory processes for specific outcomes, it may offer a more holistic view of adaptation and better reflect actual patterns of resilience occurring in real life (Masten & Powell, 2003; Schoon, 2006).

Several studies have identified child, family, and community factors that are associated with resilience to maltreatment. At the individual level, these include a higher IQ (Harpur, Polek, & van Harmelen, 2015), internal locus of control (Bolger & Patterson, 2001), good social communication (Lansford et al., 2006) and an easy temperament (Martinez-Torteya, Anne Bogat, von Eye, & Levendosky, 2009). The role of gender in resilience to childhood maltreatment is inconsistent in the literature (Chandy, Blum, & Resnick, 1996; McGloin & Widom, 2001; Samplin, Ikuta, Malhotra, Szeszko, & Derosse, 2013) and dependent on which outcomes are used to define resilience.

At the family level, protective factors include positive parent relationships (Herrenkohl, Herrenkohl, Rupert, Egolf, & Lutz, 1995), lower parent psychopathology and substance misuse (Graham-Bermann, Gruber, Howell, & Girz, 2009; Jaffee et al., 2007) and supportive non-parental family caregivers (Werner, 1997).

Community-level variables have been relatively less investigated than individual and family factors (Afifi & Macmillan, 2011; Haskett, Nears, Ward, & McPherson, 2006), however cohesive neighborhoods (Riina, Martin, & Brooks-Gunn, 2014), involvement in faith based groups (Herrenkohl et al., 2005) and school engagement (Williams & Nelson-Gardell, 2012), have been associated with resilience to childhood maltreatment. Success in the school environment is a known predictor of social and mental health functioning (Stipek, 1997) and studies have shown an association between satisfaction with school and later academic and behavioral outcomes (Goodman & Gregg, 2010; Perkins & Jones, 2004; Pharris, Resnick, & Blum, 1997). Extracurricular activities in schools also provide opportunities to set goals, exercise independence and connect to other peers (Peck, Roeser, Zarrett, & Eccles, 2008) and positive effects on exam success and psychological adjustment have been documented (Fredricks & Eccles, 2006; Goodman & Gregg, 2010).

There are very few longitudinal studies of resilience following childhood maltreatment in the UK. A better understanding of how young children who have experienced maltreatment show later successful adaptation can provide valuable information for intervening and preventing negative consequences. Using data from a large prospective UK community sample, this study sought to identify protective influences associated with different domains of competence following parental maltreatment in early life. A person-centered approach was used to identify the key factors that enable children who have been emotionally or physically maltreated by a parent before the age of 5 to achieve high educational qualifications and develop positive emotional health in adolescence.

## 2 Methods

### 2.1 Participants

The sample comprised participants from the Avon Longitudinal Study of Parents and Children (ALSPAC), an ongoing UK cohort study. Pregnant women resident in the former Avon Health Authority in south-west England, having an estimated date of delivery between 1/4/91 and 31/12/92 were invited to take part, resulting in a cohort of 14,541 pregnancies and 14,062 live births (Boyd et al., 2013; Fraser et al., 2013). Of the 13,978 singleton/twin offspring alive at one year, a small number of participants withdrew consent (n = 24) leaving a baseline sample of 13,954. Detailed information about ALSPAC is available on the study website (http://www.bristol.ac.uk/alspac), which includes a fully searchable dictionary of available data (http://www.bris.ac.uk/alspac/researchers/data-access/data-dictionary). Ethical approval for the study was obtained from the ALSPAC Law and Ethics committee and local research ethics committees.

### 2.2 Exposure to maltreatment

The study sample for these analyses was defined by maternal reports of physical or emotional maltreatment towards the child, perpetrated by either the mother or her partner. The exposure period chosen was the first 5 years of life for a number of reasons: (1) the risk of maltreatment is greatest in early childhood; (2) this period is most critical in shaping future health and development; and (3) young children are particularly vulnerable to negative influences (Irwin, Siddiqi, & Hertzman, 2007; Sidebotham, 2000; Wildeman et al., 2014). There were too few cases of sexual abuse reported to be considered in the analyses.

Data were collected from mothers using postal questionnaires at 8, 21, 33, 47 and 61 months. Approximately 10% of mothers did not respond to any questionnaires so their children could not be classified as experiencing / not experiencing maltreatment.

A child was considered emotionally or physically maltreated and included in the analysis sample if the mother reported emotional or physical cruelty towards the child *at any point*, since the focus was on exposure to any maltreatment and it was assumed unlikely that a mother would falsely report an episode of socially undesirable behavior. Partial responders were included provided at least one positive response was given. Children who were reported as not experiencing maltreatment at all of the 5 time points were excluded from the sample as according to the mother they were not maltreated in early childhood. In addition, children who were reported as not experiencing maltreatment in at least 1 questionnaire but fewer than 5, with missing data for the other time-points were excluded. Here we cannot be certain that maltreatment did not occur at these missing time-points but there is no evidence from the data that maltreatment actually happened.

**Fig. 1:**
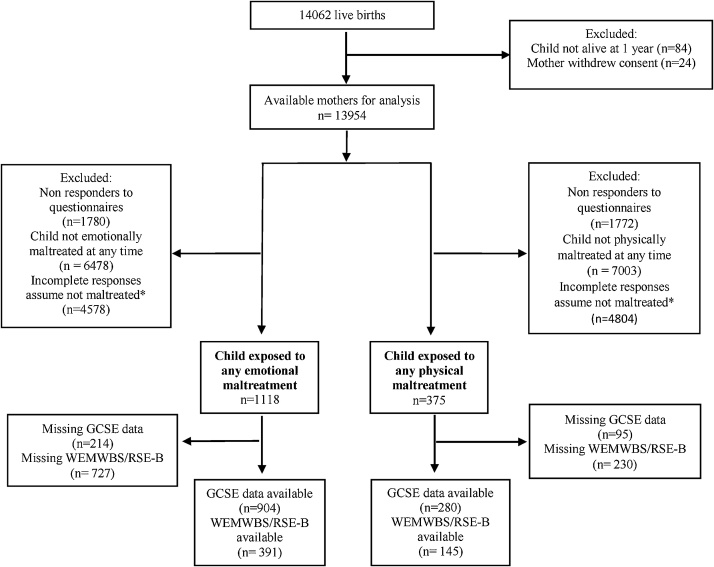
Study diagram of flow of participants in the study. WEMWBS: Warwick-Edinburgh Mental Wellbeing Score, RSE-B: Bachman Self-Esteem Scale, GCSE: General Certificate of Secondary Education. Note: * Incomplete responses refer to mothers who reported that their children did not experience maltreatment in at least one questionnaire but had missing data for the other assessments in the first 5 years of the child’s life.

The final study sample was 1118 emotionally maltreated children and 375 physically maltreated children (See Fig. 1 for full details on sample flow).

### 2.3 Outcomes

This study focused on 3 positive outcomes in adolescence, with successful adaptation following early life maltreatment assessed in terms of educational attainment, self-esteem and wellbeing.

#### 2.3.1 Educational attainment

ALSPAC data for each adolescent were linked to the National Pupil Database (NPD). This is a governmental census database published by the Department of Education, providing high quality data on pupil level attainment in state funded schools in England (Department for Education, 2015). Educational achievement was assessed using national examination results from the General Certificate of Secondary Education (GCSE), compulsory exams taken at 16 years in a range of different subjects. The number of grade A*-C GCSE exam grades achieved was chosen as the outcome. Positive adaptation in education was demonstrated by 5 or more grade A*-C GCSEs, an established performance measure of educational attainment in the UK (Harrowell, Hollén, Lingam, & Emond, 2018).

#### 2.3.2 Self-esteem and wellbeing

Adolescents’ emotional health was assessed by self-report questionnaires at age 17.5 years. Wellbeing was measured using the Warwick-Edinburgh Mental Wellbeing Scale (WEMWBS), a positively worded 14 item scale with 5 response categories summed into a single score ranging from 14 to 70 (Warwick Medical School, 2017). Self-esteem was measured using the Bachman revision of the Rosenberg's Self-Esteem Scale (RSE-B), a 10-item scale with 5 response categories summed into a score ranging from 0 to 40 (Ranzijn, Keeves, Luszcz, & Feather, 1998). Higher scores correspond to greater wellbeing and self-esteem respectively. Both measures have been used in different populations with good validity and reliability (Crawford et al., 2011; Ranzijn et al., 1998; Stewart-Brown, 2015). Positive adaptation was demonstrated by scores greater than the median of the non-maltreated ALSPAC cohort (median = 48 for the WEMWBS and 29 for the RSE-B).

### 2.4 Resilience factors

Potential individual, family, and community factors associated with resilience were chosen based on the previous literature. Hypothesized factors included: male gender, high IQ, internal locus of control, less emotional temperament, good social communication, close attachment to a grandparent, positive sibling interaction, good school attendance, positive opinion of school, engagement with religion, not being bullied, supportive friendships and regular participation in extracurricular activities. (See online Supplementary material Table 1 for a full description of how each protective factor was derived, including methods and times of measurement).

### 2.5 Potential confounders

Adjustment was made for many potential time-invariant confounding variables measured in early childhood. These included: sex of the child, maternal age at birth, level of parental education, family financial status (increased financial difficulties reported by the mother) and housing adequacy (household crowding with less than 1 room per person or periods of homelessness). These variables were derived from the ALSPAC Family Adversity Index (Bowen, Heron, Waylen, Wolke, & Team, 2005).

Whilst children in the study sample were all exposed to maltreatment, this may have varied in its severity. Since this variation in maltreatment is likely to have affected the probability of a positive outcome and may also be related to whether children experienced the potential resilience associated factors, adjustment was made for maternal reports of *persistent* maltreatment from the mother or her partner (continuing into later childhood up to 10 years of age), *co-occurring* physical and emotional maltreatment, and *additional exposure to interpersonal violence* between the parents in early childhood.

### 2.6 Statistical analysis

Initially, univariate associations between each potential protective factor and positive outcome (≥5 A*-C GCSEs or self-esteem and wellbeing scores above the median) were examined separately. Where there was evidence of a relationship, models were adjusted for confounders and level of maltreatment to identify the key protective factors. A final set of adjusted models was then created, one each for individual, family and community factors, where all the key protective factors within that domain from the initial step were adjusted for together, in addition to confounders and severity of maltreatment. Logistic regression was used and results are presented in odds ratios and their 95% confidence intervals. All statistical analyses were performed using Stata version 14.

#### 2.6.1 Missing data

Since analyses which include only complete cases can result in biased results, multiple imputation using chained equations was performed to impute missing data in the outcomes and covariates. This is an established statistical method to reduce attrition bias and increase the precision of estimates (Sterne et al., 2009). Fifty imputed datasets were created with a Monte Carlo error of approximately 10% to minimize sampling variability from the imputation process. All variables used in the complete case analyses were included in the imputation procedure, in addition to variables that predicted the missing values themselves and variables that influenced the process causing the missing data. Normality for continuous variables was not assumed. Results from analyses of the imputed data sets, where the bias from missing data is reduced, were similar to the complete case analyses. The imputed analyses are presented in the results below. (For analyses using complete cases only, see online Supplementary material Tables 2–3).

## 3 Results

### 3.1 Descriptive data

1118 children experienced emotional maltreatment by either parent in the first 5 years of life. Follow-up of these children was incomplete, resulting in 904 (81%) with GCSE information available at 16 years, and 391 (35%) with data on wellbeing and self-esteem at 17.5 years. 375 children experienced physical maltreatment in the first 5 years of life. Of these, 280 (75%) had GCSE information available at 16 years, and 145 (39%) provided data on wellbeing and self-esteem at 17.5 years (See Fig. 1 for sample attrition).

Children with incomplete follow-up emotional health outcomes at 17.5 years differed to those with complete data. Children who experienced emotional and physical maltreatment but did not provide wellbeing and self-esteem outcome data in adolescence were more likely to have younger mothers at birth and parents without educational qualifications. Children who experienced emotional maltreatment only but did not provide wellbeing and self-esteem outcome data were more likely to be male and have families with greater psychopathology, more substance misuse and increased housing and financial difficulties. In contrast, since education data was sourced from a national governmental database, there were fewer socioeconomic differences for maltreated children who did not provide GCSE outcomes at 16 years.

Among children who experienced emotional maltreatment, 59.0% (533/904) demonstrated educational resilience (achieving ≥5 A*-C GCSE grades) and 38.9% (152/391) and 41.9% (164/391) demonstrated resilience in self-esteem and wellbeing respectively (scores above the median for the non-maltreated cohort). For children who experienced physical maltreatment, 60.4% (169/280) demonstrated educational resilience and 36.6% (53/145) and 42.1% (61/145) showed evidence of resilience in self-esteem and wellbeing.

### 3.2 Factors associated with resilience

Odds ratios and 95% confidence intervals for each hypothesized protective factor after adjusting for confounders and severity of maltreatment are presented in Table 1, Table 2, using imputed data.

**Table 1:** Associations for emotionally maltreated children using multiple imputed data (n = 1118). Odds ratios and 95% confidence intervals are shown for each hypothesized protective factor separately after adjustment for confounders and severity of maltreatment on GCSE performance, wellbeing and self-esteem.

	GCSE success		Wellbeing		Self-esteem	
Potential protective factor	OR [95% CI]	p value	OR [95% CI]	p value	OR [95% CI]	p value
*Individual-*						
Male gender	0.85 [0.65–1.10]	0.216	1.96 [1.28–3.03]	0.002	1.58 [1.00–2.50]	0.052
High IQ	1.08 [1.07–1.10]	<0.001	1.01 [0.99–1.02]	0.262	1.01 [0.99–1.02]	0.335
Internal locus of control	2.32 [1.55–3.47]	<0.001	1.13 [0.70–1.84]	0.607	1.37 [0.90–2.09]	0.143
Good social communication	1.46 [1.25–1.70]	<0.001	1.32 [1.06–1.63]	0.013	1.24 [1.01–1.51]	0.040
Less emotional temperament	1.07 [0.91–1.26]	0.393	1.26 [1.00–1.58]	0.055	1.13 [0.93–1.39]	0.212

*Family-*						
Positive relationship with sibling/s	1.18 [0.98–1.41]	0.084	1.03 [0.81–1.30]	0.832	1.04 [0.83–1.30]	0.744
Close attachment to grandparent	1.13 [0.83–1.55]	0.445	0.94 [0.64–1.38]	0.735	0.93 [0.59–1.45]	0.730

*Community-*						
Supportive friendships	1.04 [0.88–1.24]	0.618	1.18 [0.96–1.45]	0.116	1.09 [0.91–1.31]	0.340
Not being a victim of bullying	1.09 [0.72–1.64]	0.681	2.05 [1.31–3.22]	0.002	1.57 [0.99–2.48]	0.055
Engagement with religion	1.42 [0.99–2.03]	0.058	0.83 [0.51–1.33]	0.430	0.93 [0.58–1.50]	0.769
Extracurricular activities	2.02 [1.46–2.79]	<0.001	1.58 [1.04–2.42]	0.034	1.60 [0.99–2.60]	0.056
Good school attendance	1.60 [0.90–2.85]	0.112	1.85 [0.69–4.96]	0.220	1.15 [0.49–2.70]	0.744
Positive opinion of school	1.53 [1.17–2.01]	0.002	1.26 [0.87–1.83]	0.217	1.50 [1.10–2.06]	0.012

*Note*: OR = odds ratios; CI = confidence interval.

**Table 2:** Associations for physically maltreated children using multiple imputed data (n = 375). Odds ratios and 95% confidence intervals are shown for each hypothesized protective factor separately after adjustment for confounders and severity of maltreatment on GCSE performance, wellbeing and self-esteem.

	GCSE success		Wellbeing		Self-esteem	
Potential protective factor	OR [95% CI]	p value	OR [95% CI]	p value	OR [95% CI]	p value
*Individual-*						
Male gender	0.77 [0.47–1.25]	0.285	1.37 [0.72–2.63]	0.337	3.33 [1.72–6.25]	<0.001
High IQ	1.08 [1.05–1.10]	<0.001	1.00 [0.98–1.02]	0.967	1.01 [0.99–1.03]	0.174
Internal locus of control	1.87 [0.99–3.50]	0.052	0.75 [0.33–1.67]	0.472	1.44 [0.72–2.86]	0.299
Good social communication	1.23 [0.99–1.52]	0.064	1.02 [0.74–1.39]	0.921	1.20 [0.89–1.60]	0.231
Less emotional temperament	1.02 [0.78–1.33]	0.903	1.00 [0.71–1.40]	0.989	1.02 [0.69–1.51]	0.925

*Family-*						
Positive relationship with sibling/s	1.13 [0.84–1.51]	0.428	0.93 [0.67–1.30]	0.683	0.82 [0.57–1.16]	0.253
Close attachment to grandparent	1.23 [0.69–2.20]	0.473	1.35 [0.61–2.98]	0.455	0.92 [0.40–2.12]	0.842

*Community-*						
Supportive friendships	0.86 [0.62–1.20]	0.381	1.18 [0.85–1.65]	0.323	1.32 [0.90–1.94]	0.160
Not being a victim of bullying	0.67 [0.36–1.25]	0.206	1.57 [0.72–3.42]	0.254	1.49 [0.62–3.56]	0.367
Engagement with religion	1.54 [0.53–4.48]	0.424	1.62 [0.75–3.50]	0.218	0.53 [0.24–1.19]	0.122
Extracurricular activities	1.97 [1.11–3.49]	0.021	1.94 [0.85–4.43]	0.112	1.89 [0.90–3.96]	0.093
Good school attendance	1.54 [0.53–4.48]	0.424	0.52 [0.17–1.60]	0.253	0.88 [0.25–3.06]	0.843
Positive opinion of school	1.68 [1.07–2.63]	0.023	1.61 [1.01–2.57]	0.045	1.35 [0.81–2.26]	0.243

*Note*: OR = odds ratios; CI = confidence interval.

#### 3.2.1 Emotional maltreatment

Among children who experienced emotional maltreatment (Table 1), there was evidence after adjusting of better GCSE attainment in those with a high IQ, an internal locus of control, good communication skills, regular participation in extra-curricular activities and enjoyment of school. These factors all demonstrated strong evidence for a protective effect. There was weak evidence of an association between engagement with religion and achieving **≥**5 A*- C GCSEs. Protective factors for both wellbeing and self-esteem after adjusting included being male, having good communication skills, not being a victim of bullying and extra-curricular activities. For high wellbeing specifically, having a less emotional temperament was weakly associated. For self-esteem, a positive opinion of school was strongly protective, however the importance of internal locus of control diminished after adjusting for confounders.

Overall, there was evidence that good communication skills, enjoyment of school and extracurricular activities were particularly important factors for children experiencing emotional maltreatment with overlapping positive effects on both education and emotional health, two conceptually different domains.

#### 3.2.2 Physical maltreatment

For children exposed to physical maltreatment (Table 2), similar protective factors for educational attainment were identified to those for emotionally maltreated children; high IQ, good communication skills, internal locus of control, engagement in activities and liking school; however, effects sizes were generally smaller. Fewer protective influences were identified for adolescents’ emotional health. For example, being male was the only factor associated with high self-esteem scores, while school enjoyment was weakly associated with positive wellbeing scores.

For both physically and emotionally maltreated children, there was insufficient evidence to suggest that the hypothesized family related factors were associated with positive adaptation in any of the three outcomes.

### 3.3 Adjusted models

The final set of adjusted models included all key protective factors together that were identified as being important from the previous steps and are presented in Table 3, Table 4.

**Table 3:** Final adjusted models for emotionally maltreated children using multiple imputed data (n = 1118). Odds ratios and 95% confidence intervals are shown using logistic regression on GCSE performance, wellbeing and self-esteem.

GCSE success			Wellbeing			Self-esteem		
Identified protective factor	OR [95% CI]	p value	Identified protective factor	OR [95% CI]	p value	Identified protective factor	OR [95% CI]	p value
Internal locus of control	1.49 [0.93–2.37]	0.094	Less emotional temperament	1.16 [0.89–1.50]	0.272	Internal locus of control	1.30 [0.85–2.00]	0.217
Good social communication	1.33 [1.12–1.59]	0.002	Good social communication	1.26 [0.99–1.61]	0.059	Good social communication	1.22 [1.00–1.49	0.054
High IQ	1.08 [1.06–1.10]	<0.001	Not being bullied	1.97 [1.25–3.13]	0.004	Not being bullied	1.44 [0.90–2.29]	0.127
Extracurricular activities	1.91 [1.36–2.69]	<0.001	Extracurricular activities	1.49 [0.96–2.29]	0.072	Extracurricular activities	1.48 [0.88–2.48]	0.134
Positive opinion of school	1.50 [1.14–1.98]	0.004				Positive opinion of school	1.46 [1.06–2.01]	0.020
Engagement with religion	1.39 [0.94–2.04]	0.097						

*Note*: OR = odds ratios; CI = confidence interval.

**Table 4:** Final adjusted models for physically maltreated children using multiple imputed data (n = 375). Odds ratios and 95% confidence intervals are shown using logistic regression on GCSE performance only.

GCSE success		
Identified protective factor	OR [95% CI]	p value
Internal locus of control	1.38 [0.65–2.90]	0.397
Good social communication	1.15 [0.88–1.51]	0.295
High IQ	1.07 [1.05–1.10]	<0.001
Extracurricular activities	1.85 [1.03-3.31]	0.038
Positive opinion of school	1.63 [1.03–2.57]	0.036

*Note*: OR = odds ratios; CI = confidence interval.

For children who experienced emotional maltreatment, high IQ (OR = 1.08, p < 0.001), good social communication (OR = 1.33, p = 0.002), positive perception of school (OR = 1.50, p = 0.004) and extracurricular activities (OR = 1.91, p < 0.001) were associated with good educational attainment in the fully adjusted model (Table 3). The importance of the individual related factors diminished in the final models for wellbeing and self-esteem with only school related factors remaining; not being bullied was most important for wellbeing (OR = 1.97, p = 0.004), and a positive view of school was the dominant influence for self-esteem (OR = 1.46, p = 0.02).

For children who experienced physical maltreatment, high IQ (OR = 1.07, p < 0.001), positive opinion of school (OR = 1.63, p = 0.036) and extracurricular activities (OR = 1.85, p = 0.038) were associated with educational resilience in the adjusted model (Table 4). Models could not be created for wellbeing and self-esteem for physically maltreated children due to the lack of protective influences identified.

## 4 Discussion

The results of this large longitudinal study demonstrate that many children exposed to parental maltreatment go on to achieve better than expected outcomes in adolescence, even when they have experienced more severe forms of maltreatment. The proportion of children who demonstrated resilience in education, self-esteem and wellbeing were similar between those exposed to emotional and physical maltreatment; however fewer protective factors were identified for children who were physically maltreated.

Within our framework of hypothesized protective factors, community factors relating to the school, including satisfaction with school, extracurricular activities and not being bullied, were most important for positive adaptation to childhood maltreatment. Compared to other factors, school-based covariates had important protective effects on *multiple* domains of functioning after controlling for confounders, and were the dominant factors in the final adjusted models. The importance of school-related factors is in keeping with the socioecological model of resilience which constructs resilience not as an individual trait but a process resulting from interactions between individuals and their environments, including the services available to them (Masten, 2011; Theron, Liebenberg, & Ungar, 2015; Ungar, 2013a). Ungar (2015) proposes that when stressors are particularly high, environmental factors become more critical for a person’s resilience than individual characteristics or cognitions. Our study suggests that a positive school environment has a key role in promoting educational and emotional health resilience to early life maltreatment.

Good communication was the only individual trait associated with resilience in *both* educational attainment and emotional health. Communication was measured by the Social and Communication Disorders Checklist, a sensitive measure of autistic traits assessing communication skills, prosocial behavior and emotion appraisal (Skuse, Mandy, & Scourfield, 2005). Children with better social communication may be more successful in finding non-aggressive solutions to problems in school and at forming trusting relationships, thereby enhancing their supportive network (Lansford et al., 2006). These coping mechanisms may help buffer the harmful impacts of maltreatment on self-esteem and wellbeing and more positive interactions with teachers and peers in school could support improved learning.

Within this study, being male was associated with higher self-esteem and wellbeing scores for emotionally maltreated children and higher self-esteem for physically maltreated children. This is consistent with evidence that suggests females are more susceptible to the mental health effects of adverse childhood stresses (Haatainen et al., 2003; Veijola et al., 1998). However, as with most maltreatment samples which tend to be predominantly female (DuMont et al., 2007), there was a strong attrition for males in our study at follow up in adolescence. Males remaining in our sample at 17.5 years were likely to be more motivated and may have other protective factors that promote good emotional health.

Although some studies have demonstrated that stable family environments and supportive caregivers are associated with resilience (Afifi & Macmillan, 2011; Domhardt, Münzer, Fegert, & Goldbeck, 2015; Haskett et al., 2006), we found little evidence in support of the protective effects of positive grandparent and sibling relationships for all 3 outcomes. This may be due to the way in which these constructs were measured- both sibling and grandparent relationships were assessed using maternal reports, and findings may have been different if child reports were used. Although susceptible to detection bias, childhood maltreatment may persist across generations (Child Welfare Information Gateway, 2016; Widom, Czaja, & Dumont, 2015). An abusive parent may have been treated cruelly by their own parents, which may explain why there was little evidence to suggest that grandparents exerted a beneficial influence. Additionally, positive sibling interactions may be difficult if other children within the family are being maltreated and vulnerable to the same negative consequences.

This study has a number of strengths. Our findings are based on a longitudinal prospective cohort study with a follow up of 12 years, using a large sample of maltreated children in the community. We investigated positive outcomes within two important and conceptually different domains in adolescence. We were able to adjust for multiple confounders and for severity of maltreatment based on maternal reports of chronicity, co-occurring abuse and exposure to interpersonal violence to demonstrate that successful adaptation occurred irrespective of the level of risk exposure. Using maternal reports to identify maltreatment resulted in a more representative sample of maltreated children compared to official child protection records which detect only a small proportion of cases, often the most severe (Lansford et al., 2006; Widom, 1988). Most official records also do not include children as confirmed cases until they are older due to delays in investigation and legal action (Jaffee et al., 2007). Finally, emotional abuse is the second most common reason for children needing protection from abuse in the UK (Bentley et al., 2017) and this is one of the few studies that examines this form of maltreatment specifically.

The findings should however be interpreted in the light of certain limitations. Firstly, as with most longitudinal cohorts, there was attrition regarding the self-esteem and wellbeing outcomes at 17.5 years. Whilst we attempted to minimize the impact of this using multiple imputation methods, this approach cannot remove bias completely. Secondly, as our dataset on maltreatment exposure and severity of maltreatment was derived from maternal report, this may be subject to recall and underreporting bias. However, participants completed questionnaires individually and were assured of the anonymity of their responses. We were unable to use registration on local Child Protection Registers (CPR) as an objective additional indicator of more severe maltreatment, due to extremely pronounced attrition amongst those with a CPR record.

Thirdly, we excluded children who were reported as not experiencing maltreatment on at least one assessment across the 5 years of data collection but had missing data for the other time-points. Maltreatment may have happened in any of these missing assessments and children with more missing assessments may be those with greater risk factors for poor outcomes, such as a lower socioeconomic status. We were unable to capture these potentially high-risk children in our sample and this could have affected the estimates of protective factors. Finally, compared to emotional maltreatment, we were unable to identify many protective factors for physically maltreated children for the wellbeing and self-esteem outcomes, apart from gender and a good relationship with school. Given the comparatively fewer number of physically maltreated children in our study (375 vs 1118), a larger sample size may have been needed.

### 4.1 Implications for policy, practice and future work

Our findings have important implications for policies that aim to buffer the negative consequences of early life maltreatment. Many of the protective factors in our final models are correlated with overlapping mechanisms. For example, ‘not being bullied’ and ‘satisfaction with school’ are interlinked (Tsitsika et al., 2014). Additionally, factors based on behaviors and attitudes may be indicative of wider underlying processes within families and schools which we are unable to fully measure in the models (Goodman & Gregg, 2010). As such, rather than focusing on isolated positive influences separately, policy should target areas which encapsulate multiple protective factors and their intertwined relationships together.

Our study suggests that the school environment is the most important area for policy to focus on, where there are multiple opportunities to improve both academic achievement and emotional health. For example, school-based strategies include zero-tolerance to bullying policies with a clear protocol for reporting abuse, more formal pastoral positions developed for teachers, and increased after-school programs and extracurricular activities. Such policies may enable maltreated children to feel more positive about school. Given that the key protective factors in our study were identified between 5–13 years of age, individuals exposed to maltreatment may particularly benefit from interventions provided in primary school.

Amongst professionals who care for children in schools, our results highlight that an increased awareness of protective individual traits, including communication skills and cognitive ability, is also important. Whilst certain characteristics like IQ are not easily amenable to change (Jaffee et al., 2007), recognition of vulnerable children who lack such protective traits provides the opportunity for prompt intervention and extra support.

Various pre-existing school-based programs could play an important role in promoting protective factors for maltreated children. The government-funded Extended Schools program in the UK enables schools to offer a range of extracurricular activities for children during evenings and holidays (Department of Education, 2017a). Activities include study support, sport, drama and music clubs, arts and crafts, cookery and language lessons, volunteering, as well as adult learning classes for parents and community access to school facilities. Whilst a formal National evaluation of this scheme has not been published, evaluation of a similar predecessor program demonstrated improvement on pupil attainment and social outcomes (Cummings et al., 2007). Despite the possible benefits for children experiencing maltreatment, the Extended Schools program is currently only available for selected schools in areas of significant social disadvantage (Department of Education, 2017b) and requires increased funding for greater coverage.

The Social and Emotional Aspects of Learning (SEAL) program is another government-funded scheme implemented in schools in England, which aims to support and enhance the emotional and social skills of children (Humphrey, Lendrum, & Wigelsworth, 2010). Delivery centers on three “waves of intervention,” involving (1) whole-school work; (2) small group interventions for children needing additional help; and (3) one to-one sessions (Humphrey et al., 2010, p. 6). Although studies have not specifically investigated the benefits for maltreated children, evaluation of the small group work intervention showed a measurable and sustained quantitative positive impact on teacher- and pupil- rated social skills (Humphrey et al., 2008). Given the importance of social communication in our study, similar school-based interventions have the potential to support children exposed to maltreatment. However programs need to be evaluated with proper trials to ensure empirical information on protective factors is actually translated into improved outcomes.

Given the dynamic nature of resilience (Klika & Herrenkohl, 2013; Rutter, 2012), continued follow up of our study into adulthood is needed. Future work could involve evaluating mediating factors and outcomes over repeated time-points in adulthood to understand whether the relationship between resilience and protective factors changes across time. Measures of severity of maltreatment that are not dependent on parental reports may help confirm that protective factors continue to function for children with greater harmful exposures.

## 5 Conclusion

Our findings support the literature that many children who experience maltreatment in early life achieve positive outcomes despite their adversity. As a community-based study, our findings captured the vast number of maltreated children who are not identified by the social or legal authorities and are likely to be representative of many families in the UK. For children who have been emotionally or physically maltreated by a parent, factors related to the school environment were most important in facilitating resilience. Protective effects of school went beyond academic achievement and included nurturing adolescents’ wellbeing and self-esteem. Encouragingly, many of the identified factors are not unique to maltreated children, suggesting that factors promoting resilient outcomes in adolescence are universal. Interventions based within the school setting that seek to promote wellbeing and self-esteem among all children, appear most justified. However, future work is necessary to understand how exposure to maltreatment interacts with protective factors over time and impacts on resilience across the life-course.

## Funding

The UK Medical Research Council, the Wellcome Trust (Grant ref: 102215/2/13/2) and the University of Bristol provide core support for ALSPAC. Funding which supported the collection of the primary outcomes included the Wellcome Trust and MRC (Grant ref 092,731). A comprehensive list of grants funding (PDF, 459KB) is available on the ALSPAC website.

Specific funding for this study was provided by the Severn Foundation School and the Centre for Child and Adolescent Health, University of Bristol. The funding sources had no involvement in the conduct of the research and/or preparation of the paper.

## Declaration

This publication is the work of the authors: Nisreen Khambati, Liam Mahedy, Jon Heron and Alan Emond, who will serve as guarantors for the contents of this paper. We confirm that this work is original and has not been published elsewhere, nor is it currently under consideration for publication elsewhere. We have no conflicts of interest to disclose.
