# Supplementary material for: Educational and emotional health outcomes in adolescence following maltreatment in early childhood: A population-based study of protective factors
Source: Child Abuse Negl. 2018 Jul;81:343–53. doi: 10.1016/j.chiabu.2018.05.008 (PMC6013281; doi:10.1016/j.chiabu.2018.05.008)
Supplement: Supplementary file 1 [file mmc1.docx]

# **SUPPLEMENTARY MATERIAL**

**Table 1: Hypothesized protective factors**

Full description of how each potential protective factor was derived, using a mixture of questionnaires answered by the mother or study child and face-to-face clinics. Factors were measured after the child was 5 years old (i.e. after the period when exposure to child maltreatment was assessed).

|  | Measure | Method of measurement | Type of Variable | Setting | Hypothesized protective factor |
| --- | --- | --- | --- | --- | --- |
| *Individual* | **Gender** | Gender | Binary  (female/male) | n/a | Female gender |
|  | **Cognitive function** | Short form of the Wechsler Intelligence Scale for Children III (Wechsler, 1992), an IQ test administered to the child at 8 years 6 months. | Continuous | Research clinic | High IQ |
|  | **Locus of control** | 12 item shortened version of the Nowicki-Strickland Internal-External scale for preschool and primary children (Nowicki & Duke, 1974), administered to the child at 8 years 6 months. A summary score with two levels was created by dividing by the mean. | Binary (internal/external) | Research clinic | Internal locus of control |
|  | **Temperament** | 5 item Emotionality subscale of the Emotionality Activity Sociability scale (Buss & Plomin, 1984), reported by the mother when the child was 5 years 9 months. | Continuous | Questionnaire | Less emotional temperament |
|  | **Communication** | 12-item Social and Communication Disorders checklist (Skuse, Mandy, & Scourfield, 2005), reported by the mother when the child was 7 years 7 months. | Continuous | Questionnaire | Good social communication |
| *Family* | **Grandparent relationship** | Mother asked whether child was particularly attached to grandparent when the child was 7 years 7 months. | Binary  (yes/no) | Questionnaire | Close attachment to grandparent |
|  | **Sibling interaction** | Summary score derived from 7 questions answered by the mother on the frequency the child does different activities with their siblings when the child was 11 years 8 months. Activities included playing, reading, drawing, going out, talking, eating and playing sport. | Continuous | Questionnaire | Positive sibling relationship |
| *Community* | **School attendance** | Total number of days off school the child had taken in the last year for both health & non-health reasons, answered by the mother when the child was 7 years 7 months. A summary score with two levels was created by dividing by the 90^th^ quartile (10 days off). | Binary  (≤ 10/>10 days off) | Questionnaire | High school attendance with no more than 10 days off in the last year |
|  | **Perception of school** | Summary score derived from questions answered by the child aged 14 years 1 month on how strongly they agreed with seven positively stated opinions of school using a five-point Likert scale. Common stem was “my school is a place where I…” with consequent statements: …really like to go each day, …like to be, …feel proud to be a pupil, …have a lot of fun, …enjoy what I do, …get excited about the work I do. | Continuous | Questionnaire | Positive opinion of school |
|  | **Religion** | Summary measure derived from two questions answered by the mother on whether the child engaged with religion by praying or attending a place of worship at 9 years 7 months. | Binary  (yes/no) | Questionnaire | Engagement with religion |
|  | **Bullying** | Summary measure derived from a modified version of the Bullying and Friendship Interview schedule (Wolke, Woods, Stanford, & Schulz, 2001), reported by the child aged 12 years 6 months. Questions related to the frequency of five types of overt bullying (theft, threats, physical violence, nasty names, nasty tricks), and four types of relational bullying (social exclusion, spreading lies or rumors, coercive behavior, deliberately spoiling games) in the prior 6 months. | Binary  (not bullied/bullied) | Research clinic | Not receiving any frequent direct or indirect bullying in the prior 6 months. |
|  | **Friendship** | Summary score derived from five questions based on a shortened version of the Friendships  questionnaire from the Cambridge Hormones and Moods Project (Goodyer, Wright, & Altham, 1990), answered by the child age 12 years 6 months. Questions related to the child’s overall satisfaction with friends and the number of friends, frequency the child sees friends outside school, whether the child can talk to friends about problems & whether friends are understanding. | Continuous | Research clinic | Supportive friendships |
|  | **Extracurricular activity** | Summary measure derived from two questions answered by the mother on whether the child  regularly attended classes/clubs for extracurricular activities like sports, dance etc. or attended special groups like beavers, scouts when the child was 6 years 9 months. | Binary  (yes/no) | Questionnaire | Regular participation in extracurricular activities |

**Table 2: Univariate associations for emotionally maltreated children (n=1118) using the complete case data set only**

Odds ratios and 95% confidence intervals are shown for each hypothesized protective factor separately after adjustment for confounders and severity of maltreatment on GCSE attainment, wellbeing and self-esteem.

|  | GCSE success | | | | Wellbeing | | | Self-esteem | | |
| --- | --- | --- | --- | --- | --- | --- | --- | --- | --- | --- |
| Potential protective factor | | ***n*** | ***OR [95% CI]*** | ***p value*** | ***n*** | ***OR [95% CI]*** | ***p value*** | ***n*** | ***OR [95% CI]*** | ***p value*** |
| *Individual-* | | | | | | | | | | |
| Female gender | | 876 | 1.23 [0.93-1.62] | 0.140 | 383 | 0.47 [0.31-0.72]  # | 0.001 | 383 | 0.54 [0.35-0.82] | 0.004 |
| High IQ | | 537 | 1.09 [1.07-1.11] | < 0.001 | 311 | 1.01 [1.00-1.03] | 0.169 | 311 | 1.01 [0.99-1.02] | 0.225 |
| Internal locus of control | | 523 | 3.22 [2.05-5.05] | < 0.001 | 306 | 1.30 [0.80-2.11] | 0.289 | 306 | 1.58 [0.98-2.57] | 0.062 |
| Good social communication | | 632 | 1.44 [1.24-1.68] | 0.001 | 327 | 1.33 [1.06-1.65] | 0.013 | 327 | 1.27 [1.01-1.59] | 0.040 |
| Less emotional temperament | | 675 | 1.14 [0.97-1.35] | 0.113 | 341 | 1.41 [1.12-1.79] | 0.004 | 341 | 1.26 [1.00-1.58] | 0.049 |
| *Family-* | | | | | | | | | | |
| Positive relationship with sibling/s | | 518 | 1.26 [1.05-1.52] | 0.015 | 291 | 1.01 [0.79-1.28] | 0.967 | 291 | 1.09 [0.85-1.39] | 0.501 |
| Close attachment to grandparent | | 635 | 1.29 [0.90-1.85] | 0.171 | 328 | 0.94 [0.59-1.50] | 0.807 | 328 | 0.94 [0.59-1.49] | 0.788 |
| *Community-* | | | | | | | | | | |
| Supportive friendships | | 506 | 1.03 [0.85-1.24] | 0.789 | 317 | 1.25 [1.00-1.58] | 0.055 | 317 | 1.22 [0.97-1.54] | 0.094 |
| Not being a victim of bullying | | 499 | 0.96 [0.62-1.47] | 0.854 | 314 | 2.64 [1.56-4.46] | <0.001 | 314 | 1.82 [1.08-3.06] | 0.024 |
| Engagement with religion | | 504 | 1.48 [0.98-2.22] | 0.063 | 283 | 0.79 [0.48-1.30] | 0.353 | 283 | 0.77 [0.47-1.27] | 0.306 |
| Extracurricular activities | | 667 | 2.25 [1.59-3.17] | < 0.001 | 342 | 1.66 [1.00-2.74] | 0.048 | 342 | 1.81 [1.09-3.01]  \| | 0.022 |
| Good school attendance | | 634 | 1.30 [0.68-2.49] | 0.423 | 325 | 2.28 [0.77-6.78] | 0.139 | 325 | 1.10 [0.41-2.95] | 0.844 |
| Positive opinion of school | | 355 | 1.67 [1.23-2.27] | 0.001 | 247 | 1.26 [0.89-1.77] | 0.188 | 247 | 1.60 [1.11-2.31] | 0.011 |

Note: OR= odds ratios; CI = confidence interval

**Table 3: Univariate associations for physically maltreated children (n=375) using the complete case dataset only**

Odds ratios and 95% confidence intervals are shown for each hypothesized protective factor separately after adjustment for confounders and severity of maltreatment on GCSE attainment, wellbeing and self-esteem.

.

|  | GCSE success | | | Wellbeing | | | | Self-esteem | | | |
| --- | --- | --- | --- | --- | --- | --- | --- | --- | --- | --- | --- |
| Potential protective factor | ***n*** | ***OR [95% CI]*** | ***p value*** | ***n*** | ***OR [95% CI]*** | ***p value*** | ***n*** | | | ***OR [95% CI]*** | ***p value*** |
| *Individual-* | | | | | | | | | | | |
| Female gender | 274 | 1.22 [0.74-2.01] | 0.429 | 145 | 0.58 [0.29-1.15] | 0.122 | 145 | | 0.36 [0.18-0.74] | | 0.006 |
| High IQ | 189 | 1.09 [1.06-1.12] | <0.001 | 124 | 1.00 [0.98-1.02] | 0.803 | 124 | | 1.02 [1.00-1.05] | | 0.052 |
| Internal locus of control | 186 | 3.35 [1.58-7.12] | 0.002 | 120 | 1.30 [0.58-2.88] | 0.524 | 120 | | 2.92 [1.17-7.29] | | 0.022 |
| Good social communication | 216 | 1.26 [0.99-1.61] | 0.057 | 126 | 1.07 [0.80-1.43] | 0.654 | 126 | | 1.37 [0.99-1.88] | | 0.057 |
| Less emotional temperament | 223 | 1.11 [0.82-1.50] | 0.502 | 135 | 1.16 [0.78-1.72] | 0.455 | 135 | | 1.34 [0.88-2.04] | | 0.171 |
| *Family-* | | | | | | | | | | | |
| Positive relationship with sibling/s | 182 | 1.31 [0.94-1.83] | 0.110 | 115 | 0.97 [0.65-1.45] | 0.888 | 115 | | 0.67 [0.42-1.05] | | 0.077 |
| Close attachment to grandparent | 217 | 1.39 [0.74-2.62] | 0.308 | 126 | 1.45 [0.69-3.06] | 0.328 | 126 | | 1.18 [0.54-2.57] | | 0.685 |
| *Community-* | | | | | | | | | | | |
| Supportive friendships | 176 | 0.82 [0.58-1.16] | 0.271 | 124 | 1.17 [0.83-1.65] | 0.366 | 124 | | 1.33 [0.91-1.94] | | 0.135 |
| Not being a victim of bullying | 176 | 0.65 [0.31-1.39] | 0.268 | 123 | 2.39 [1.03-5.54] | 0.042 | 123 | | 2.54 [1.04-6.19] | | 0.041 |
| Engagement with religion | 179 | 1.36 [0.68-2.71] | 0.384 | 112 | 1.20 [0.54-2.67] | 0.652 | 112 | | 0.42 [0.18-1.00] | | 0.050 |
| Extracurricular activities | 220 | 2.17 [1.18-3.99] | 0.013 | 131 | 2.06 [0.90-4.70] | 0.086 | 131 | | 2.04 [0.85-4.89] | | 0.109 |
| Good school attendance | 216 | 1.23 [0.40-3.80]  \| | 0.715 | 123 | 0.48 [0.12-1.98] | 0.312 | 123 | | 1.14 [0.25-5.12] | | 0.865 |
| Positive opinion of school | 126 | 2.10 [1.14-3.87] | 0.018 | 95 | 1.59 [0.91-2.80] | 0.106 | 95 | | 1.76 [0.97-3.19] | | 0.062 |

Note: OR= odds ratios; CI = confidence interval

**REFERENCES**

Buss, A., & Plomin, R. (1984). *Temperament : Early developing personality traits*. Hillsdale, New Jersey: Lawrence Erlbaum.

Goodyer, I., Wright, C., & Altham, P. (1990). Recent achievements and adversities in anxious and depressed school age children. *J Child Psychol Psychiatry, 31*(7), 1063-1077.

Nowicki, S., & Duke, M. (1974). A preschool and primary internal-external control scale. *Developmental Psychology, 10*(6), 874-880.

Skuse, D. H., Mandy, W. P., & Scourfield, J. (2005). Measuring autistic traits: heritability, reliability and validity of the Social and Communication Disorders Checklist. *Br J Psychiatry, 187*, 568-572.

Wechsler, D. (1992). *Wechsler Intelligence Scale for Children – Third Edition UK Manual*. Sidcup: The Psychological Corporation.

Wolke, D., Woods, S., Stanford, K., & Schulz, H. (2001). Bullying and victimization of primary school children in England and Germany: prevalence and school factors. *Br J Psychol, 92*(Pt 4), 673-696.
